# The complete genome sequence of *Listeria monocytogenes* strain S2542 and expression of selected genes under high-pressure processing

**DOI:** 10.1186/s13104-021-05555-2

**Published:** 2021-04-15

**Authors:** Ilhan Cem Duru, Florentina Ionela Bucur, Margarita Andreevskaya, Anne Ylinen, Peter Crauwels, Leontina Grigore-Gurgu, Bahareh Nikparvar, Tone Mari Rode, Pia Laine, Lars Paulin, Trond Løvdal, Christian U. Riedel, Nadav Bar, Daniela Borda, Anca Ioana Nicolau, Petri Auvinen

**Affiliations:** 1 Institute of Biotechnology, University of Helsinki, Helsinki, Finland; 2 Faculty of Food Science and Engineering, Dunarea de Jos University of Galati, Galati, Romania; 3 Department of Chemical Engineering, Norwegian University of Science and Technology (NTNU), Trondheim, Norway; 4 Department of Process Technology, Nofima–Norwegian Institute of Food, Fisheries and Aquaculture Research, 4068 Stavanger, Norway; 5 Institute of Microbiology and Biotechnology, Ulm, University, Albert-Einstein-Allee 11, 89081 Ulm, Germany; 6 Present Address: Blueprint Genetics, Espoo, Finland

**Keywords:** High-pressure processing, Genome comparison, Listeria, Food safety, Gene expression, Stress recovery, Foodborne pathogen

## Abstract

**Objectives:**

The study aims to generate the whole genome sequence of *L. monocytogenes* strain S2542 and to compare it to the genomes of strains RO15 and ScottA. In addition, we aimed to compare gene expression profiles of *L. monocytogenes* strains S2542, ScottA and RO15 after high-pressure processing (HPP) using ddPCR.

**Results:**

The whole genome sequence of *L. monocytogenes* S2542 indicates that this strain belongs to serotype 4b, in contrast to the previously reported serotype 1/2a. Strain S2542 appears to be more susceptible to the treatment at 400 MPa compared to RO15 and ScottA strains. In contrast to RO15 and ScottA strains, viable cell counts of strain S2542 were below the limit of detection after HPP (400 MPa/8 min) when stored at 8 °C for 24 and 48 h. The transcriptional response of all three strains to HPP was not significantly different.

**Supplementary Information:**

The online version contains supplementary material available at 10.1186/s13104-021-05555-2.

## Introduction

*Listeria monocytogenes* is a gram-positive foodborne bacterium that can cause severe infections in humans. Listeriosis, the associated disease, particularly affects individuals with compromised immune systems [1] and may lead to hospitalization and mortality rates of 20–30% [2]. Humans are generally infected following consumption of contaminated ready-to-eat (RTE) food products that do not undergo thermal treatment during the manufacturing process or are contaminated post-thermal treatment. *L. monocytogenes* can thrive in a range of inhospitable environmental conditions including low temperatures thus causing significant challenge to the food industry [3–6].

Recently, we have studied the transcriptional response of *L. monocytogenes* strains RO15 and ScottA to HPP by RNA-seq [7, 8]. We observed that our previous gene expression results [8] are negatively correlated with the results of a previous study on HPP-induced changes in gene expression of *L. monocytogenes* strain S2542 [9]. Thus, our aim was to make a draft assembly of the genome of the strain S2542 (obtained from Tasmanian Institute of Agricultural Research) and to compare the genome sequences of these three strains.

Based on the conflicting results regarding the HPP-induced changes in gene expression, we also performed a new set of HPP experiments under the same conditions as in our previous study [8] with all the three strains, and analyzed the transcriptional response of a number of representative genes.

## Methods

### *L. monocytogenes* strain S2542 DNA isolation, library preparation, sequencing, assembly, and comparative genomics

Genomic DNA of *L. monocytogenes* strain S2542 [9] was isolated as described previously [7]. A library for sequencing of the genome was generated using Nextera XT kit according to the instructions from the manufacturer (Illumina, San Diego, CA, USA). The obtained library was paired-end sequenced on a MiSeq platform using a 600 cycle sequencing kit v3 (Illumina).

Quality trimming and nextera adapter removal was done using Cutadapt v1.14 [10] with -q 25 and -m 50 options. Trimmed reads were assembled using SPades v3.13.0 [11] with default options. Prokka v1.13.3 [12] was used for functional annotation. A secondary functional annotation was done using PANNZER2 [13]. Serotype of the strain S2542 was predicted by aligning the serotype marker primers [14] to the genome using EMBOSS primersearch v 6.6.0 [15]. Multilocus sequence typing based on 7 loci (MLST), clonal complex (CC), and lineage was predicted by uploading the genome to BIGSdb-Lm webserver (https://bigsdb.pasteur.fr/listeria/listeria.html) [16]. Multiple genome alignment for strains RO15, ScottA and S2542 was performed using Mauve v2.4.0 [17]. Blast Average Nucleotide Identity (ANIb) analysis between strains was done using JSpeciesWS webtool [18]. Core genome alignment was done using Roary v3.12.0 [19] with the “-mafft” option for the same strains that we used previously [7]. Phylogenetic tree was created from the alignment using FastTree v2.1.11 [20].

## HPP experiments

HPP experiments were conducted in order to compare the gene expression profiles of RO15, ScottA and S2542 strains. The strains were cultivated as described previously [8] and pressurized at 400 MPa, 8 °C for 8 min. Viability of *L. monocytogenes* cells was determined after storage of the samples at 8 °C for 0 min, 24 h and 48 h by performing serial decimal dilutions of both treated samples and corresponding controls in phosphate buffered-saline solution (PBS; Sigma Aldrich, St. Louis, SUA; pH 7.4) and plating dilutions on BHI (brain heart infusion) agar plate. Colony forming units were counted after incubation of the plates at 37 °C for 48 h.

## RNA extraction

RNA was extracted from HPP-treated samples and the controls with NucleoSpin RNA kit (Macherey-Nagel, Düren, Germany) as described previously [7].

## ddPCR

ddPCR was used to compare gene expression levels between strains RO15, ScottA, and S2542. Three replicate samples for each treatment and strain, and their corresponding control samples were analyzed. Expression levels of seven genes (*recG, fusA, clpE, hly, agrB, ftsE*, and *mscL)* were quantified using strains RO15, ScottA, and S2542 samples treated with 400 MPa and recovered for 0 h or 24 h. Primers (Additional file 1: Table S1) were designed using Primer3Plus [21] and manufactured by Integrated DNA Technologies. The protocol used, including gDNA removal and RT–PCR steps, was performed as described previously [22]. To be able to compare expression levels of different samples, expression of the target genes (cDNA copies/µl) was normalized using concentrations of two stably expressed genes (*recG* and *fusA*). To allow comparison to our previously published RNA-Seq data, the results were expressed as log_2_ (gene concentration in treated sample/gene concentration in control sample) values.

## Results and discussion

### Genome assembly of *L. monocytogenes* strain S2542

We sequenced and assembled the genome of strain S2542 and compared it with the genomes of the previously studied strains; RO15 and ScottA. Assembly and annotation resulted in a 2.9 Mbp genome consisting of 14 contigs (N50 = 477482 bp) with 2839 predicted coding sequence (CDS). GC-content of strain S2542 was 37.9%, which is identical to the GC-content of ScottA. Based on Blast Average Nucleotide Identity (ANIb), the genome of strain S2542 was more similar to ScottA than to RO15 (ANIb scores S2542/ScottA: 99.97, S2542/RO15: 94.55). Genome alignment of strains RO15, ScottA, and S2542 indicated high similarities between the genomes. Ortholog gene prediction between three strains revealed that 31 genes were present in S2542 but absent in strains ScottA and RO15 (Additional file 1: Table S1). Most of the genes specific to strain S2542 were annotated to encode hypothetical proteins (Additional file 1: Table S1). In total, 49 genes were found in both ScottA and RO15 but not in S2542, and 47 of these were prophage genes (Additional file 1: Table S1). Thus, the differences in genome sequence most likely do not explain the differences in gene expression response to HPP described in our previous study on RO15 and ScottA and study presented for strain S2542 [8, 9].

Based on PHASTER prophage prediction, two small regions in S2542 (Contig1:183471-206364: 22.8 kb; Contig2: 99648-114057: 14.4 kb) were annotated as a prophage with a low confidence score. Homolog of these regions were seen in both strain RO15 and ScottA. We did not observe CRISPR–Cas or anti-CRISPR genes within the strain S2542 genome.

**Fig. 1:**
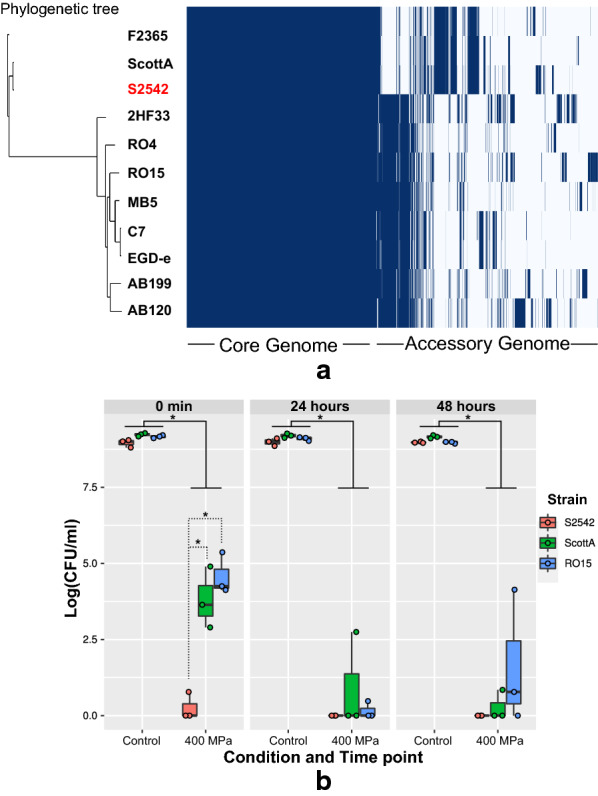
**a** Pan-genome visualization of selected strains, **b** viability (Log(CFU/ml)) of *L. monocytogenes* strain S2542, ScottA, and RO15 before and after the pressure treatment 400 MPa, 8 min. **a** Phylogenetic tree have been calculated using the core genomes of the indicated strains. *L. monocytogenes* strain names were shown at the end of the phylogenetic tree branch. The matrix shows presence (blue) and absence (white) of the core and strain-specific genes. **b** Effect of HPP on cell survival. 0 min represents samples plated on agar just after pressure treatment, 24 h and 48 h represent samples that were allowed to recover for 24 h and 48 h at 8 °C and plated on agar. Strain S2542 is presented in red, ScottA in green, and RO15 in blue. For each experimental group individual values of three biological replicates (HPP of independent bacterial cultures) and box-and-whiskers with median and 25th–75th percentile are shown. Data was analysed by pairwise Student’s t-test and differences between groups were considered statistically significant at p-values < 0.05 (indicated by *)

Based on genome sequence analysis using BIGSdb [16], multilocus sequence typing (based on seven loci) assigned strain S2542 to ST-145. In addition, based on genome sequence, the clonal complex (CC) of strain S2542 was CC2 and it belongs to the Lineage I, as ScottA. Serotype prediction based on the genome suggested that strain S2542 belongs to PCR-serogroup 4b. By contrast S2542 was reported to belong to serogroup 1/2a based on an antigen test [9]. Pan-genome analysis showed that strain S2542 harboured similar genes as serotype 4b strains (Fig. 1a). Core genome alignment tree visualization also supported assignment of S2542 to serotype 4b, since two clear clusters were observed for serotype 4b strains (F2365, ScottA, S2542) and serotype 1/2a strains (2HF33, RO4, RO15, MB5, C7, EGD-e, AB199, AB120) (Fig. 1a.)

### Viable cell count after HPP

To assess cell recovery capacity after HPP, all three strains were treated at 400 MPa and 8 °C for 8 min and colony forming units (CFU) were enumerated immediately after treatment and after storage for 24 and 48 h at 8 °C (Fig. 1b). For all three strains CFU/ml were higher for the samples plated immediately after HPP (t = 0 min) compared to the samples stored for 24 and 48 h. Also, the reduction in CFU/ml at t = 0 min after HPP compared to untreated controls was significantly higher for S2542 than for ScottA and RO15. Moreover, after 24 h and 48 of storage no viable cells could be detected for S2542, i.e. CFU/ml for all replicates of the high pressure treated samples were below the limit of detection, whereas for ScottA and RO15 CFU were detected at least for some of the replicates at these timepoints. We previously predicted that certain phage genes, CRISPR–Cas system and anti-CRISPR genes might play a role in high-pressure resistance based on pan-genome analysis [7]. Strain S2542 does not harbour CRISPR–Cas system, nor anti-CRISPR genes. This might be one of the reasons for higher susceptibility to HPP in strain S2542.

### HPP-induced changes in expression of representative genes

In order to investigate the contradictory results of HPP-induced gene expression between the Bowman et al. [9] study and our previous results [8], a number of genes that showed strong negative correlation between the two studies were selected and their expression levels were analysed by ddPCR approach. In contrast, we did not observe any difference in the HPP-induced expression changes of the selected genes between these strains. Furthermore, gene expression changes at 400 MPa after 24 h for strains ScottA and S2542 were significantly correlated (Pearson correlation; r = 0.97) (Table 1). This indicates that the gene expression responses of strains S2542 and ScottA are similar in these experimental conditions. No significant differences were observed between RO15 and S2542 (Table 1). Thus, expression profiles of the selected genes in response to HPP appear to be not significantly different between the strains. The contradictory results between our previous RNA-seq study [8] and the microarray and qPCR data reported by Bowman et al. [9] are difficult to explain, but could be potentially explained by the different experimental conditions (8 °C–8 min HPP exposure in Duru et al. [8] and 15 °C–5 min HPP exposure in Bowman et al. [9]) or difference of the growth of the bacteria during the experiments.

**Table 1:** Comparison of two studies [8, 9] and ddPCR-based expression changes for strain RO15, ScottA and S2542

(a) log_2_ fold change of the indicated gene after HPP at 400 MPa as determined by RNA-seq for RO15 and ScottA (Duru et al. [8]) or microarray for S2542 (Bowman et al. [9])			
Gene	RO15 (RNA-seq, Duru et al. [8]) log_2_ fold change	ScottA (RNA-seq, Duru et al. [8]) log_2_ fold change	S2542 (microarray, Bowman et al. [9]) log_2_ fold change
*agrB*	− 0.35	− 0.15	2.84
*hly*	0.89	2.57	− 4.34
*ftsE*	− 1.11	− 0.66	2.71
*clpE*	2.26	2.27	− 3.93
*mscL*	0.24	0.33	1.04
Pearson correlation with S2542	− 0.87	− 0.99	

(b) log_2_ fold change of the indicated gene immediately after HPP at 400 MPa as determined by ddPCR for all three strains (this study)			
Gene	RO15	ScottA	S2542
*agrB*	− 0.10	− 0.16	− 0.32
*hly*	− 0.02	0.34	0.36
*ftsE*	− 0.30	− 0.06	0.20
*clpE*	0.27	0.27	0.57
*mscL*	0.01	− 0.73	0.83
Pearson correlation with S2542	0.49	− 0.24	

(c) log_2_ fold change of the indicated gene immediately after HPP at 400 MPa and storage for 24 h as determined by ddPCR for all three strains (this study)			
Gene	RO15	ScottA	S2542
*agrB*	0.36	0.09	0.59
*hly*	− 0.06	0.08	0.45
*ftsE*	0.02	0.60	1.78
*clpE*	0.28	0.21	0.35
*mscL*	0.57	0.82	2.30
Pearson correlation with S2542	0.38	0.97	

**a** log_2_ fold change results of the two different studies [8, 9] with samples treated at 400 MPa and correlation of the RNAseq results to the DNA microarray results. **b** ddPCR log_2_ fold change results at 0 min after 400 MPa treatment comparison with data from strains RO15, ScottA and S2542 showing the correlation of the gene expression between them. **c** ddPCR log_2_ fold change results at 24 h after 400 MPa treatment with data from strains RO15, ScottA, and S2542 showing the correlation of the gene expression between them

The correlation of our previous RNA-seq results [8, Accession number: PRJEB34771] and current ddPCR results were also investigated. Log_2_ fold changes of selected genes were correlated (Pearson correlation; r = 0.95 and 0.64 for strain RO15 and ScottA, respectively) for both strains at 0 min timepoint. However, at the 24 h timepoint, no clear correlation was observed for the selected genes. This indicates gene expression levels immediately after HPP (0 min timepoint) were consistent between two different experiments [8 and this study], but after a long incubation period (24 h timepoint) gene expression levels differed.

## Conclusion

In conclusion, we provide the whole draft genome sequence of *Listeria monocytogenes* strain S2542. The genome sequence revealed that strain S2542 belongs to serotype 4b, although it has previously been reported to belong to serotype 1/2a. Analysis of viable cell count at different timepoints after HPP suggests that S2542 is more sensitive to HPP than ScottA or RO15. Previous results of two studies on HPP-induced gene expression in S2542 on the one hand and RO15 and ScottA on the other hand were in disagreement [8, 9]. Here, we conducted transcriptional analysis comparing the HPP-induced changes of all three strains directly. The results suggest that despite differences in sensitivity and recovery from HPP, none of the strains respond significantly differently to HPP, at least under the conditions tested and for the genes analyzed.

## Limitations

In the present study, the transcriptional response of five different genes were compared between three different strains of *L. monocytogenes*. Although genes were carefully selected and representative for two previous studies, it is possible that the global gene expression response of S2542 may still be different than that of RO15 and/or ScottA.

## Supplementary Information

**Additional file 1: Table S1**. The list of orthologous genes within strains and primers that were used in ddPCR experiments.

## Data Availability

All sequencing data and assembled genome have been deposited in the European Nucleotide Archive (ENA) under Accession code PRJEB42816. For pan-genome analysis, we publicly available genome assemblies of *L. monocytogenes* strain ScottA (https://www.ncbi.nlm.nih.gov/assembly/GCF_000212455.1), strain RO15 (https://www.ncbi.nlm.nih.gov/assembly/GCF_902827145.1), strain EGDe (https://www.ncbi.nlm.nih.gov/assembly/GCF_000196035.1), strain 2HF33 (https://www.ncbi.nlm.nih.gov/assembly/GCA_902838845.1), strain C7 (https://www.ncbi.nlm.nih.gov/assembly/GCA_902837585.1), strain MB5 (https://www.ncbi.nlm.nih.gov/assembly/GCA_902838455.1), strain RO4 (https://www.ncbi.nlm.nih.gov/assembly/GCA_902833935.1), strain AB120 (https://www.ncbi.nlm.nih.gov/assembly/GCA_902837535.1), strain AB199 (https://www.ncbi.nlm.nih.gov/assembly/GCA_902837525.1), and strain F2365 (https://www.ncbi.nlm.nih.gov/assembly/GCF_000008285.1) from NCBI database.

## Abbreviations

HPP: High pressure processing
ddPCR: Digital droplet PCR
MLST: Multilocus sequence typing
CC: Clonal complex
